# Representation of Asian American Populations in Medical School Curricula

**DOI:** 10.1001/jamanetworkopen.2022.33080

**Published:** 2022-09-23

**Authors:** Peter Sang Uk Park, Eda Algur, Sweta Narayan, William B. Song, Matthew D. Kearney, Jaya Aysola

**Affiliations:** 1 Perelman School of Medicine, University of Pennsylvania, Philadelphia; 2 Department of Family Medicine and Community Health, Perelman School of Medicine, University of Pennsylvania, Philadelphia; 3 Penn Medicine Center for Health Advancement, Perelman School of Medicine, University of Pennsylvania, Philadelphia; 4 Office of Inclusion, Diversity, and Equity, Perelman School of Medicine, University of Pennsylvania, Philadelphia; 5 Leonard Davis Institute of Health Economics, University of Pennsylvania, Philadelphia

## Abstract

**Importance:**

Despite being one of the fastest-growing populations in the US, the Asian American population is often misrepresented in and omitted from health research and policy debate. There is a current lack of understanding of how Asian American populations are portrayed in medical school curricula.

**Objective:**

To assess how Asian American populations and their subgroups are represented in medical school curricula.

**Design, Setting, and Participants:**

In this qualitative study, the content of 632 lectures from all 19 courses of the preclinical curriculum at a single US institution from the academic year 2020 to 2021 was analyzed to identify and characterize unique mentions of race and ethnicity as well as granular ethnicity. Among the 632 lectures, we identified 256 nonrepetitive, unique mentions of race and ethnicity or granular ethnicity. These unique mentions were coded and analyzed for emerging patterns of use.

**Main Outcomes and Measures:**

Study outcomes included (1) the frequency of specific racial and ethnic categories mentioned in the curriculum, (2) the relative proportion of mentions of race and ethnicity that involved or included Asian American data by courses and context, and (3) key themes representing emerging patterns found from qualitative analysis of curriculum content for mentions of Asian American populations or lack thereof.

**Results:**

Among the 632 lectures, 256 nonrepetitive mentions of race and ethnicity or granular ethnicity were identified; of these, Asian American populations and/or their subgroups were mentioned in 79 of the instances (30.9%). The most common terms used to denote Asian American populations were *Asian*, with 36 mentions (45.6%); followed by *Japanese*, with 10 mentions (12.7%); and *Chinese*, with 8 mentions (10.1%). Overall, there were 26 mentions (10.2%) of American Indian or Alaska Native populations, 12 mentions (4.7%) of Asian and Pacific Islander or Asian American and Pacific Islander populations, 67 mentions (26.2%) of Asian or Asian American populations, 143 mentions (55.9%) of Black or African American populations, 62 mentions (24.2%) of Hispanic or Latino populations, 4 mentions (1.6%) of Native Hawaiian or Pacific Islander populations, and 154 mentions (60.2%) of White populations. During the analysis of the curriculum for representation of Asian American populations, the following 5 key themes emerged from the data: (1) omission, (2) aggregation, (3) inconsistent categorization, (4) misidentification of granular ethnicity, and (5) association of race and ethnicity with disease.

**Conclusions and Relevance:**

This qualitative study suggests that the curriculum from a single US medical school largely mirrors the inappropriate use of race and ethnicity found in published health literature and clinical guidelines. Solutions with long-term results will require collaboration among diverse groups of interest to adopt inclusive research programs and design. Such solutions could better equip students in combating race-based medicine and could promote community outreach programs built based on trust.

## Key Points

**Question:**

How are Asian American populations represented in medical school curricula?

**Findings:**

This qualitative study of a curriculum from 1 US medical school revealed 5 key themes from discussions about Asian American populations in the context of race- and ethnicity-related data in health literature: (1) omission, (2) aggregation, (3) inconsistent categorization, (4) misidentification of granular ethnicity, and (5) association of race and ethnicity with disease.

**Meaning:**

Findings of this study suggest that medical school curricula reflect the inappropriate use of race and ethnicity found in published health literature and clinical guidelines.

## Teaser

This qualitative study analyzes the content of 632 lectures from all 19 preclinical courses at a single US medical school to assess how Asian American populations and their subgroups are represented in medical school curricula.

## Introduction

The Asian American population is one of the fastest growing racial and ethnic populations in the US, composed of more than 20 diverse subgroups with different historical backgrounds, socioeconomic characteristics, languages, cultures, and values.^1,2,3,4^ Despite their growth and heterogeneity, Asian American populations and their subgroups remain underrepresented in health research, rendered invisible in health policies and discussion.^5,6,7,8^ Moreover, small sample sizes, inadequate questionnaire design, and data aggregation contribute to misrepresentation of Asian American populations and their subgroups even when they are included.^3,9^ These challenges limit our ability to identify and address inequities in health and health care for Asian American populations.

As training grounds for the next generation of physicians, medical schools are uniquely positioned to help address racial and ethnic inequities within the health care system. Unfortunately, the misuse of race and ethnicity without proper context remains embedded in medical curricula, not only reflecting the inherent biases and faults of the medical field but also propagating them.^10,11,12,13^ Although previous recommendations addressing the misrepresentation of race and ethnicity in medicine have been published,^10,14,15,16^ there remains a paucity of studies on the representation of Asian American populations and their subgroups within medical school curricula. Therefore, in this study, we examined all the lecture slides and accompanying notes of the entire preclinical medical curriculum of a single institution to understand how medical education represents the health and health care of Asian American populations. Furthermore, we tied our findings to the theoretical framework of Asian Critical Theory, a subset of Critical Race Theory, to analyze them within the broader historical and theoretical context and racialization of the Asian American experience.

## Methods

### Data Collection

**Figure 1.**
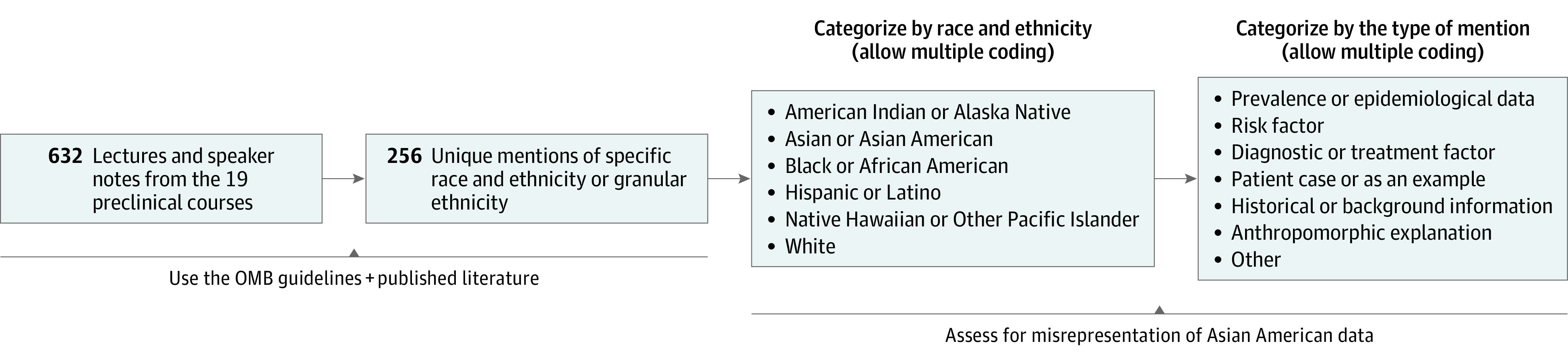
Flowchart of Methods for Assessing Race and Ethnicity and Mentions of Asian American Populations OMB indicates Office of Management and Budget.

We screened all 632 lecture slides with or without speaker notes from the 19 courses in the 2020 to 2021 preclinical curriculum at a single US medical school to identify 256 nonrepetitive mentions of specific racial and ethnic groups or granular ethnicities. Audio recordings were not transcribed. Figure 1 outlines our process of flagging unique mentions by context. To avoid data conflation, we deemed a mention as unique if it was not repeated in the same lecture slide or speaker notes. When Asian American populations were not mentioned but 2 or more other racial and ethnic groups were, we reviewed the relevant literature to evaluate whether Asian- or Asian American–specific data and research were available. This qualitative study was deemed as exempt from review and the requirement for informed consent by the University of Pennsylvania institutional review board because it was determined to not qualify as human participants research. The study protocol adhered to the Standards for Reporting Qualitative Research (SRQR) reporting guideline.

### Coding and Data Analysis

We used the categories of race and ethnicity outlined by the US Census Bureau—American Indian or Alaska Native populations, Asian or Asian American populations, Black or African American populations, Hispanic or Latino populations, Native Hawaiian or Pacific Islander populations, and White populations—along with Office of Management and Budget guidelines^17^ and published literature^14,18,19,20^ to quantify representation. We further differentiated when Pacific Islander populations were combined with the Asian or Asian American group as Asian and Pacific Islander populations or Asian American and Pacific Islander populations. eTable 1 in the Supplement details all terms that were mentioned and categorized into racial and ethnic categories. Informed by previous work,^10,12^ we characterized the context for each mention in the curriculum. For example, we noted whether *Asian American* was mentioned as an identifier for a patient case vs mentioned in the context of presenting disease prevalence by race and ethnicity (ie, summary of epidemiologic data). If a single mention had multiple contexts, we coded all of them. We examined each mention for emerging patterns to develop and iteratively refine a codebook with clear definitions and representative examples.

Of the 256 unique mentions, a subset of 50 mentions was jointly analyzed to ensure coding accuracy and assess preliminary intercoder reliability. eTable 2 in the Supplement provides further details of the categories and representative examples of the context of the mentions. The remaining 206 mentions were divided to be independently coded by 4 medical students (P.S.U.P., E.A., S.N., and W.B.S.) of the study team. Each member coded a unique set of 45 mentions plus a shared sample of 25 or 26 mentions for assessment of intercoder reliability. All coding discrepancies were resolved by group consensus. We used REDCap, version 12.5.4 (Vanderbilt University) for coding^21^ and Excel, version 16.63.1 (Microsoft Corp) for all data management. Facilitated by Stata/SE software, version 15 (StataCorp LLC), we assessed intercoder reliability using the Holsti coefficient,^22^ and determined agreement to be acceptable among coders (mean, 0.95 [range, 0.80-1.00]; median, 0.97).

## Results

### Sample Characteristics

**Figure 2.**
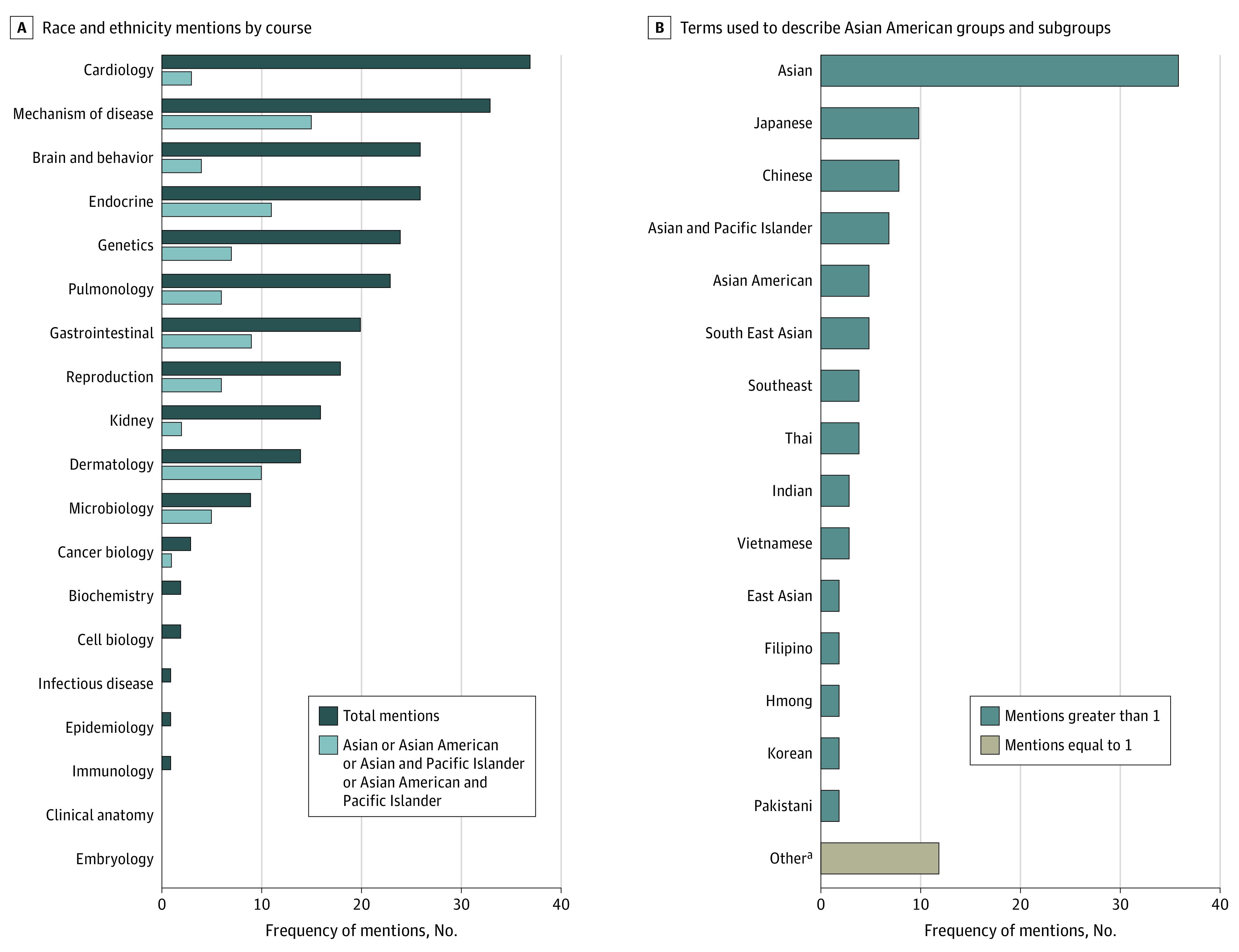
Frequency of Mentions of Asian American Populations and Race and Ethnicity Detailed overview of the frequency of mentions of Asian American populations and race and ethnicity by course (A) and term (B) used for the 79 of 256 mentions (30.9%) that refer to Asian American groups and/or their subgroups. ^a^Includes Asian American and Pacific Islander, Chinese Han, East and South Asian American, Japanese American, Karen, Laotian, Malaysian, non-Hispanic Asian or Pacific Islander, North Asian, North East Asian, “Philipino,” and Taiwanese populations.

Of the 256 mentions of 2 or more racial and ethnic groups in the analyzed sample of 632 lectures, we found a total of 79 mentions (30.9%) of Asian American populations in different forms across 19 courses. Mentions of Asian American populations were most frequent in the following courses: dermatology (10 of 14 [71.4%]), followed by microbiology (5 of 9 [55.6%]) and mechanism of disease (15 of 33 [45.5%]). Of the 79 mentions of Asian American populations, the most common term was *Asian*, comprising 36 mentions (45.6%); followed by *Japanese*, with 10 mentions (12.7%); and *Chinese*, with 8 mentions (10.1%). Figure 2 details the frequency of race and ethnicity and Asian American mentions by course and of each term used to denote Asian American populations.

**Figure 3.**
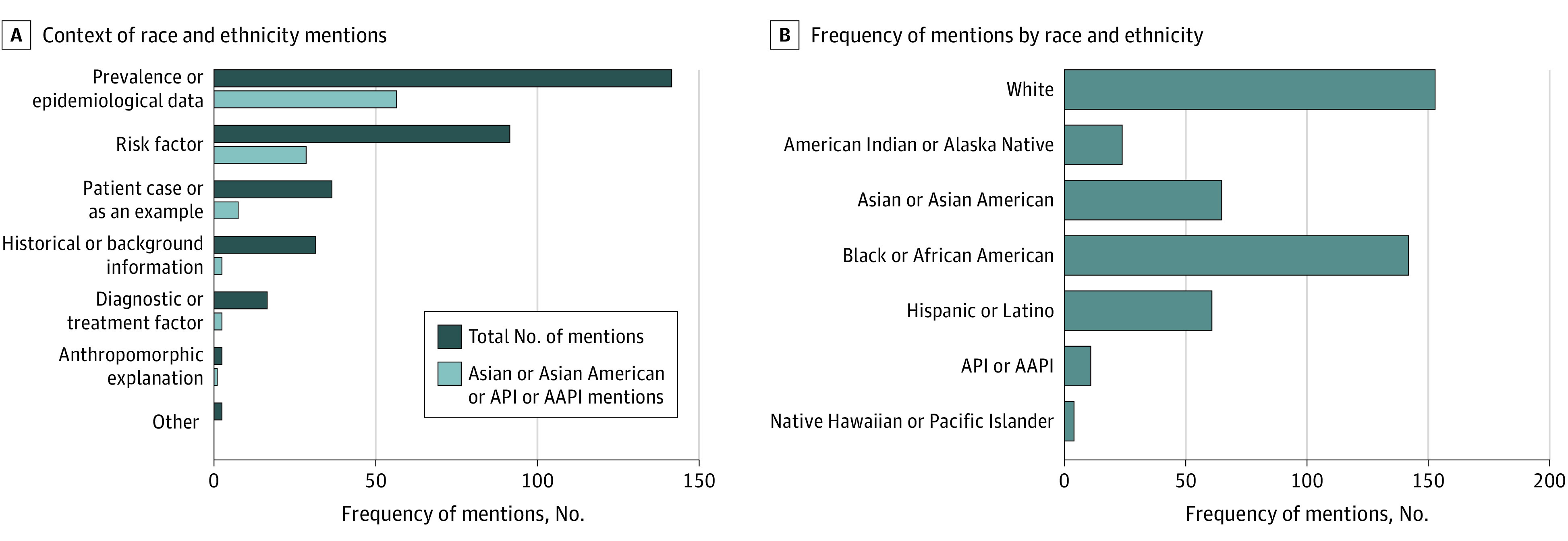
Context and Frequency of Mentions of Race and Ethnicity The frequency of mentions of Asian American populations and race and ethnicity mentions by context (A) and frequency (B). The mentions were allowed to be coded to multiple categories. AAPI indicates Asian American and Pacific Islander; and API, Asian and Pacific Islander.

Of the 256 unique mentions of race and ethnicity, 142 (55.5%) were categorized as prevalence or epidemiologic data, 92 (35.9%) as a risk factor for disease or pathologic finding, 37 (14.5%) as a patient case or clinical example, 32 (12.5%) as historical or background information, 17 (6.6%) as a factor associated with diagnosis or treatment, 3 (1.2%) as an anthropomorphic explanation for variation in physical traits, and 3 (1.2%) as other categories (Figure 3A). Of the 79 mentions of Asian American populations, 57 (72.2%) discussed epidemiologic data.

Overall, there were 26 mentions (10.2%) of American Indian or Alaska Native populations, 12 mentions (4.7%) of Asian and Pacific Islander or Asian American and Pacific Islander populations, 67 mentions (26.2%) of Asian or Asian American populations, 143 mentions (55.9%) of Black or African American populations, 62 mentions (24.2%) of Hispanic or Latino populations, 4 mentions (1.6%) of Native Hawaiian or Pacific Islander populations, and 154 mentions (60.2%) of White populations within the total 256 mentions (Figure 3B).

Five key themes emerged that characterize the patterns of representation of Asian American populations within the medical school’s curriculum: (1) omission, (2) aggregation, (3) inconsistent categorization, (4) misidentification of granular ethnicity, and (5) association of race and ethnicity with disease. Table 1 describes each theme and provides representative examples.

**Table 1.** Key Themes of Misrepresentation of Asian or Asian American Populations in Medical School Curricula

Misrepresentation	Description	Representative examples
Omission	Exclusion of Asian American populations or their subgroups in presentations of health or epidemiologic data of ≥2 racial and ethnic groups	Teaching students the varying incidence of cervical cancer in different racial and ethnic groups with a graph that only includes “All races,” “Black (including Hispanic),” “Hispanic (any race),” and “Non-Hispanic White”
Aggregation	Presenting data as a single group to mask meaningful distinctions between Asian American subgroups	Reporting the incidence of type 2 diabetes among US adults using the Asian category even though Asian American subgroups have varying rates of incidence according to publicly available disaggregated data
Inconsistent categorization	Lack of standardization or consistency in language used to describe Asian American populations and their subgroups	Interchanging *Chinese* with *East Asians*; using various terms, such as *Asian*, *Asian and Pacific *Islander* (API)*, and *East and South Asian*, throughout different lectures
Misidentification of granular ethnicity	Misassigning an Asian American subgroup to a granular ethnicity that does not match its country of origin	Presenting a hirsutism scoring table that incorrectly assigns a study population from Korea to the ethnicity of Chinese
Association of race and ethnicity with disease	Use of descriptions or strategies that explicitly or implicitly associate a specific race and ethnicity with a disease	Emphasizing the Asian ancestry of patients in numerous clinical vignettes involving thalassemia without further context, even when the information is unnecessary for diagnosis or interpretation

### Key Themes

#### Omission

When curriculum content mentioned 2 or more racial and ethnic groups but excluded Asian American populations or their subgroups, we categorized their representation as omission.^7^ White populations constituted 154 of the 256 mentions of race and ethnicity (60.2%) and Black or African American populations constituted 143 mentions (55.9%). All other racial and ethnic groups received less than half as many mentions. One example was a graph illustrating the incidence of non-Hodgkin lymphoma in only 2 racial and ethnic categories of White and Black populations, excluding data for Asian American populations as well as for Hispanic or Latino populations and American Indian or Alaska Native populations.

Throughout the curriculum, most content mentioned at least 2 other racial and ethnic groups (177 [69.1%]), but did not include Asian American populations or their subgroups. Omission rates varied between courses. Cardiology had the greatest overall unique mentions of racial and ethnic data (n = 37), but only 3 mentions (8.1%) of Asian American populations. In contrast, mentions of Asian American populations accounted for 10 of 14 mentions of race and ethnicity (71.4%) in a dermatology course.

#### Aggregation

Aggregation was coded when data from different Asian American subgroups were grouped into a single panethnic entity.^7^ An example is mention of the prevalence of chronic hepatitis B infection among Asian American populations in aggregate without discussion of the variation in prevalence within Asian countries.

#### Inconsistent Categorization

The lack of standardization or consistency in language used to describe Asian American populations and their subgroups was coded as inconsistent categorization. For instance, different terms such as *Asian*, *Asian and Pacific Islander*, and *East and South Asian* were used to denote Asian American populations. Inconsistent terminology also resulted in erroneous conflation of terms such as *Chinese* with *East Asians*.

#### Misidentification of Granular Ethnicity

Misidentification is the misassignment of an Asian American subgroup to a granular ethnicity that does not match its country of origin. We found 2 instances of misidentification, a unique pattern. The first instance was in a table summarizing data from a review article^23^ about the Hirsutism Scoring System. Although the table correctly reports that 1 of the studies analyzed in the review article took place in Korea,^24^ it describes the study population as Chinese. Similarly, the second instance involved a prevalence chart that groups individuals from urban Mauritius and Taiwan into the Chinese population. The origin of the prevalence chart could not be determined owing to lack of citation.

#### Association of Race and Ethnicity With Disease

Association of race and ethnicity with disease occurred when the curricular content used descriptions or strategies that explicitly or implicitly made an association between a specific race and ethnicity and a disease or condition without providing sufficient context or a mechanism.^10^ In the curriculum, the principal mechanism by which Asian American race and ethnicity became associated with disease conditions was repetition. A representative example involved clinical vignette cases discussing thalassemia that emphasized the Asian American heritage of the patients as Vietnamese, Chinese, Thai, and/or Indian without further context.

## Discussion

We analyzed more than 600 lecture slides across 19 courses to assess how the curriculum at a single US medical school represented data about Asian American populations and their health. Five key themes emerged in our analysis: omission, aggregation, inconsistent categorization, misidentification of granular ethnicity, and association of race and ethnicity with disease.

Misrepresentation of Asian American populations in higher education has been previously examined through the lens of Asian Critical Theory.^2,25,26^ The central tenet of this theory is the racialization of Asian American populations into monolithic stereotypes, such as the “overachieving model minorities” and “perpetual foreigners” as a form of oppression.^25^ The model minority myth attributes the economic success of all members of Asian American populations to hard work and cultural values.^27^ This myth minimizes the role of discrimination experienced by Asian American populations and maintains White supremacy by suggesting that other minoritized groups are responsible for inequities in opportunity or advancement.^28^ Meanwhile, the perpetual foreigner label marginalizes Asian American populations as peripheral to US society and questions the legitimacy of members of Asian American populations as US citizens. These stereotypes influence societal narratives of Asian American populations and affect their representation or lack thereof in research and higher education.^26^ The omission of Asian American data in medical school curricula may reflect these stereotypes and the general invisibility of Asian American data in research.^8,29^ Omitting data on Asian American populations fails to educate physicians-in-training on the implications of both health care disparities and risk for disease among omitted groups^7^—discussing cardiovascular disorders, for example, without reference to Asian American populations despite cardiovascular disorders being the leading cause of mortality among nearly all Asian American subgroups.^9^

Although aggregation of Asian American populations by panethnic terms could be a powerful signature of political alliance as seen during the US Civil Rights movement, it can also lead to imprecise statistics and racial and ethnic erasure without proper intentionality and framework.^28,30^ Gogue et al^30^ describe the disappointment and trauma some Pacific Islander populations feel in spaces for Asian and Pacific Islander populations that are not inclusive of the experiences of Pacific Islander populations. In addition, previous studies have shown that Native Hawaiian and Pacific Islander populations are at a higher risk than other Asian American groups for COVID-19 mortality^31^ and chronic health conditions, such as cardiovascular disease, owing to substantial health and socioeconomic disparities.^3,5^ Considering the small size of the Native Hawaiian and Pacific Islander community, aggregation of this high-risk group into categories such as Asian or Asian American and Pacific Islander populations substantially underestimates disease burden in this community and impedes progress in mitigating health disparities.^3,5,7^ In alignment, we use the term *Asian American* instead of *Asian American*,* Native Hawaiian*,* and Pacific Islander* to refer to those of Asian descent in the US.

Aggregation of Asian American populations as single monoliths in medical research and education further perpetuates the model minority myth of Asian American populations as a healthy and well-adjusted group, undermining diversity and health equity. For instance, disaggregation of Asian American medical school applicants, who are often considered overrepresented, has previously revealed a lack of representation in various subgroups, such as Indonesian, Cambodian, and Japanese populations.^32^ Moreover, the discussion of type 2 diabetes among Asian American populations in aggregate neglects the varying prevalence of type 2 diabetes within subgroups, which ranges from 12.6% among Asian Indian populations to 5.6% among Chinese populations.^33^ In addition, the relative lack of mentions of Asian Indian populations further reflects the perspective centered on populations considered to be East Asian and marginalization of other minoritized Asian American subgroups often encompassed by the aggregated discussion of Asian American populations.^2,30^ Aggregation in this way serves to foster clinicians’ lack of knowledge of Asian American health and the risk of underdiagnosing disease.^34,35^

Inconsistent terminology, present throughout the curricula, increases the risk for extrapolation—generalizing the findings derived from an Asian American subgroup to a broader category.^7^ Conflating Chinese populations with East Asian populations may incorrectly transpose findings specific to Chinese populations onto those of Japanese or Korean descent. This inconsistency reflects broader discrepancies in the social construction of racial and ethnic groups, which differ across societies based on historical and political factors, not biological ones. For example, the UK historically represents Asian Indian and Pakistani as a separate racial and ethnic category distinct from Asian, a practice not seen in the US. The use of granular ethnicity, as recommended by a 2009 National Academy of Medicine report, may help eliminate many of these discrepancies.^17^

Misidentification of one Asian American subgroup as another not only represents an inaccurate presentation of data but also a disregard for the unique heritage of individual Asian American subgroups. Analogous to the miscategorization of a person to a group to which they do not belong, misidentification of individuals is considered a form of microaggression with implications for wellness.^36,37,38^

Association of race and ethnicity with disease in medical education can occur in subtle ways, priming learners to unconsciously adopt race- and ethnicity-biased diagnostic and clinical guidelines.^10^ For example, discussing high rates of chronic hepatitis B infection among Asian American populations masks the substantial variation within Asian countries in which they are located, such as a prevalence of 13.6% in Laos vs 5.3% in South Korea, and overlooks immigration from endemic regions as a cause of high infection rates among minoritized groups.^39,40^ Linking disease conditions with race and ethnicity subgroups in medicine not only encourages race bias in clinical reasoning but also downplays differences between more granular ethnic groups.

Themes of misrepresentation found in this study are not unique to Asian American representation. Other racial and ethnic groups, including Latino or Hispanic Americans, were similarly underrepresented in our curriculum. Mentions of American Indian and Alaska Native populations were negligible, a phenomenon that has previously been described as *statistical genocide*.^41^ Addressing the misuse of race and ethnicity in medicine should promote advocacy efforts focused on collaboration and allyship across all minoritized groups.^42^

### Recommendations

This study revealed key mechanisms by which a single medical school curriculum misrepresented Asian American data. In many circumstances, the lecture slides and speaker notes reflected the misuse of race and ethnicity commonly found in published health literature. Instead of maintaining a passive stance, medical schools should proactively cultivate antiracist curricula^43^ for their students, who will eventually apply such medical knowledge in clinical practice, teach or lead fields with that knowledge, or generate new medical knowledge as physician scientists.

Several initiatives have been led by both students and faculty to address the misuse of race in the curricula. Work from Amutah et al,^10^ for instance, provides recommendations that guide course directors in identifying and correcting for the misuse of race and ethnicity. However, the present analysis reveals that neglected areas stem from challenges with representation of Asian American data not merely in how medical knowledge is imparted, but rather in how our medical knowledge is generated and associated with the portrayal of Asian American populations in society at large. Table 2 summarizes our short- and long-term recommendations to counter the 5 ways curricula misrepresent data about Asian American populations.^5,6,7,10,12,14,15,16,17,44,45,46,47,48^

**Table 2.** Short-term and Long-term Recommendations for Improving the Representation of Asian American Race and Ethnicity

Misrepresentation	Short-term recommendations	Long-term recommendations
Omission	When presenting racial and ethnic data, use the most comprehensive and inclusive data available When noninclusive data must be used, provide a rationale and state limitations (Shah and Kandula^6^ 2020)	Include Asian American populations and their subgroups in research study designs and avoid the classification of Asian American populations as “other” (Holland and Palaniappan^7^ 2012) Increase funding for regional and local studies inclusive of Asian American data, such as the California Health Interview Survey (Srinivasan and Guillermo^5^ 2000) Increase health care workforce diversity (Obra et al^44^ 2021) Reduce the stigma of research participation for Asian American patient populations and barriers to participation, such as language (Obra et al^44^ 2021)
Aggregation	Report disaggregated epidemiologic data when available, especially if granular differences are known (Srinivasan and Guillermo^5^ 2000, Shah and Kandula^6^ 2020, Holland and Palaniappan^7^ 2012) Assume differences in subgroups exist until proven otherwise (Shah and Kandula^6^ 2020)	Oversample Asian American subgroups in research studies to avoid aggregating data for statistical power(Srinivasan and Guillermo^5^ 2000, Shah and Kandula^6^ 2020, Holland and Palaniappan^7^ 2012) Partner and collaborate with organizations invested in and trusted by Asian American communities to increase research participation (Holland and Palaniappan^7^ 2012, Ghosh^45^ 2010)
Inconsistent categorization	Use consistent language (Flanagin et al^46^ 2021) across the lecture and notes to avoid extrapolation Establish a standardized list of racial and ethnic categories (Amutah et al^10^ 2021, Ulmer et al^17^ 2009) that can be adopted by the lecturers	Implement journal publishing and research guidelines that encourage the authors and investigators to use precise racial and ethnic categories (Flanagin et al^46^ 2021, Kaplan and Bennett^47^ 2003)
Misidentification of granular ethnicity	Include citations and original resources to the lecture materials discussing race and ethnicity to determine the origin of the misidentification (Krishnan et al^16^ 2019)	Set standards for medical schools to identify and rectify the misuse of race and ethnicity in the curriculum (Nieblas-Bedolla et al^15^ 2020)
Association of race and ethnicity with disease	Avoid statements or questions that associate a single ethnicity with a particular condition Identify and remove any unsupported use of race and ethnicity as a risk factor in lectures and textbooks (Sheets et al^48^ 2011)	Eliminate questions that award racial bias and heuristics in standardized examinations such as the United States Medical Licensing Examination (Amutah et al^10^ 2021, Ripp and Braun^14^ 2017, Nieblas-Bedolla et al^15^ 2020) Discourage the use of race and ethnicity as the proxy for genetic variation in research studies (Amutah et al^10^ 2021, Tsai et al^12^ 2016, Kaplan and Bennett^47^ 2003)

### Limitations

This study has some limitations. The first is the inherent fault in attempting to categorize various granular ethnic groups into the 6 racial and ethnicities defined by the US Census Bureau and Office of Management and Budget guidelines. The concepts and terminology surrounding race and ethnicity are constantly evolving, and certain groups may not fit precisely into arbitrarily defined racial and ethnic categories.^49^ We also did not examine the racial and ethnic diversity of the lecturers, which could be further associated with representation within the curricula. Another limitation is the restriction of the study to a single institution; investigation at different medical schools may yield varying results based on the epidemiology of the region where they are located. Despite this limitation, the present study reveals the lack of Asian American representation in the research that our medical school relied on to teach their students. Focusing on a single institution also allowed for greater precision and granularity in our analysis. Furthermore, this study serves as a reflective reminder for other medical schools to examine the inclusivity of their own curricula and provides generalizable insights for institutions to identify and correct for specific patterns of misrepresentation of Asian American populations.

## Conclusions

In this qualitative study, content analysis of more than 600 medical school preclinical curriculum slides from a single US instiution revealed 5 key themes that characterize representation of Asian American populations in medical education: (1) omission, (2) aggregation, (3) inconsistent categorization, (4) misidentification of granular ethnicity, and (5) association of race and ethnicity with disease. Although these issues may be partially mitigated by making direct modifications to medical curricula, solutions with long-lasting results will require conscious, collaborative efforts among scientific, educational, and service organizations to better train future leaders of medicine to recognize and criticize race- and ethnicity-based clinical medicine and promote inclusive research representative of diverse populations.

## Notes

Supplement. **eTable 1.** Mentioned Terms by Race and Ethnicity **eTable 2.** Context, Description, and Representative Examples of Mentions of Race and Ethnicity
