# Supplementary material for: Representation of Asian American Populations in Medical School Curricula
Source: JAMA Netw Open. 2022 Sep 23;5(9):e2233080. doi: 10.1001/jamanetworkopen.2022.33080 (PMC9508660; doi:10.1001/jamanetworkopen.2022.33080)
Supplement: Supplement. — eTable 1. Mentioned Terms by Race and Ethnicity eTable 2. Context, Description, and Representative Examples of Mentions of Race and Ethnicity [file jamanetwopen-e2233080-s001.pdf]

Supplementary Online Content

Park PSU, Algur E, Narayan S, Song WB, Kearney MD, Aysola J. Representation of Asian American populations in medical school curricula. *JAMA Netw Open.* 2022;5(9):e2233080. doi:10.1001/jamanetworkopen.2022.33080

eTable 1. Mentioned Terms by Race and Ethnicity
eTable 2. Context, Description, and Representative Examples of Mentions of Race and Ethnicity

This supplementary material has been provided by the authors to give readers additional information about their work.

© 2022 Park PSU et al. *JAMA Network Open.*

eTable 1. Mentioned Terms by Race and Ethnicity

Race and Ethnicity	Mentioned Terms
American Indian or Alaska Native (Includes South American Indians)	American Indian/Alaska Native, Alaska Native, American Indian, Peruvian Indians, Peruvian Incas, Choctaw Native Americans, Pima, Native Americans, Quechua, Chile Mapuche, Yanomano Indians, Eskimos, Aleuts, Non-Hispanic American Indian or Alaska Native
Asian and Pacific Islander or Asian American and Pacific Islander	Asian / Pacific Islander, Asian American and Pacific Islanders, Non-Hispanic Asian or Pacific Islander
Asian or Asian American	Asian-American, Southeast Asians, Asians, Japanese, East Asian, South Asian, Indians, Thais, "Philipinos", Vietnamese, Chinese, Korean, Karen, Filipino, East and South Asian American, Chinese Han, Japanese American, Taiwanese, Malaysian, Laotian, Pakistani, North Asian, North East Asian
Black or African American	African American, African, Sub-Saharan, African, Non-Hispanic Black, Black, Black South or Central American, Black Caribbean, Black African, Afro-Caribbean, African Caribbean, Black Americans
Hispanic or Latino	Hispanic, Hispanic American, Latino, Latinx, Latino-American, Caucasian/Hispanic, Latina, Latin American, Mexican American, Hispanic or Latino, Brown, Spanish
Native Hawaiian or Pacific Islander	Hawaiian or Pacific Islander, Pacific Islander, Aboriginal Australians, New Guineans
White	Mediterranean, European, Ashkenazi Jewish, European American, White, Non-Hispanic White, Caucasian, Northern European, Greeks, Cypriots, Turks, Sardinians, White European, Middle Eastern or North African, Western, Middle Eastern, Caucasian/Hispanic, Scandinavian, Celtic, Nova Scotia, English, French, German, Canadian, Egyptian

© 2022 Park PSU et al. *JAMA Network Open.*

**eTable 2.** Context, Description, and Representative Examples of Mentions of Race and Ethnicity		
Context of Mention	Description	Representative Examples
Prevalence or epidemiological data	Table, graph, or statements that illustrate proportion of individuals with a particular condition	Table outlining the prevalence of psoriasis in different racial and ethnic groups
Risk factor	Explicitly or implicitly describing race and ethnicity as a factor that increases the probability of an adverse event or condition	Listing race and ethnicity of African Americans, Hispanic Americans, Native Americans, Asian Americans, and Pacific Islanders as a major risk factor for Type 2 Diabetes
Patient case or as an example	Clinical scenarios involving specific person(s) and or their family members	Case of a 23-year-old female with Family History of “Indian” in case discussing thalassemia
Historical or background information	Information that is historical in nature without reference to internal biological factors	Descriptions of how “Spanish colonists” in 17th century observed “Peruvian Indians using the bark of the cinchona tree to treat malaria”
Diagnostic or treatment factor	Listing a particular race and ethnicity as a criterion for making a diagnosis, treatment decisions, or clinical management	Mentioning that African American patients are “most responsive” to thiazide diuretics without any further explanation
Anthropomorphic explanation	Quantitative or qualitative assessment of a race and ethnic group using physical body measurements such as size/content of muscle and fat	Stating that “Asian populations expand subcutaneous fat less and have visceral fat at lower Body Mass Index (BMI)”
Other	Mentions that did not fit in the main contexts above	Mentions of racial correction in electrocardiogram screening criteria, elimination of race consideration in estimated glomerular filtration rate, blood pressure reduction through Black barbershops

© 2022 Park PSU et al. *JAMA Network Open.*
